# Research progress on the pathophysiological mechanisms of pioglitazone-induced heart failure: a review

**DOI:** 10.3389/fcvm.2026.1843086

**Published:** 2026-09-18

**Authors:** Jianli Li, Yifei Zhang, Xuewen Li

**Affiliations:** Department of Cardiology, Third Hospital of Shanxi Medical University, Shanxi Bethune Hospital, Shanxi Academy of Medical Sciences, Tongji Shanxi Hospital, Taiyuan, Shanxi, China

**Keywords:** clinical risk prediction, fluid retention, heart failure, insulin sensitizers, pathological cardiac remodeling, pioglitazone, thiazolidinediones

## Abstract

Pioglitazone is thought to increase the risk of heart failure exacerbation in vulnerable individuals, primarily through fluid retention and plasma volume expansion. However, the precise molecular and cellular mechanisms remain incompletely understood. This narrative review aims to collect, organise, and evaluate current evidence on the pathophysiological mechanisms driving pioglitazone-related heart failure. We categorised preclinical and clinical data into key pathophysiological domains and graded evidence strength as high, moderate, or limited. The key domains implicated in this process include renal fluid retention, direct myocardial effects, altered energy metabolism, vascular changes, neurohormonal activation, and individual genetic susceptibility. While fluid retention and vascular changes demonstrate strong clinical evidence, direct myocardial toxicity remains primarily observed *in vitro* and lacks clinical confirmation. Neurohormonal activation and metabolic disturbances are supported by moderate evidence from animal models and cohort studies, whereas evidence for genetic susceptibility and direct cardiotoxicity remains limited. Overall, this review grades the strength of current evidence and provides a framework for the safe clinical use of pioglitazone and the design of targeted risk prevention strategies.

## 1 Introduction

Pioglitazone is a thiazolidinedione (TZD) and a very effective peroxisome proliferator-activated receptor gamma (PPAR*γ*) agonist that has been used to control type 2 diabetes mellitus (T2DM) for a long time by increasing insulin sensitivity and maintaining normal blood glucose levels (1). In addition to lowering blood glucose, this agent can also protect beta-cell function to maintain a healthy level of blood sugar over time, improve the control of cardiovascular risk factors and other surrogate indicators of cardiovascular disease, etc (2). However, since its use in clinics, there have been many debates and deep studies on whether pioglitazone increases the risk of heart failure; therefore, its application in clinical practice has been restricted (3, 4).

It should be noted that the clinical data supporting a mechanism of pioglitazone-associated heart failure in vulnerable patients mainly point to volume overload, and direct cardiotoxicity has largely been observed *in vitro* with little clinical translation (5, 6). At the same time, in this review, we explicitly distinguish between evidence from clinical trials, animal models and *in vitro* studies to help readers determine the strength of each proposed mechanism.

Early in the clinical course, the first sign of harm was an increase in fluid retention; thus, this likely worsened the heart failure (7). Several studies have shown that there are varying results for this risk in patients. For example, a large-scale meta-analysis of prospective clinical trials found that although pioglitazone can reduce the risk of major adverse cardiovascular events (MACE) and stroke, it also increases the risk of heart failure and heart failure-related hospitalisation (4). A general introduction to glucose-lowering drugs was also provided, and an overview of the agent associated with an increased risk of heart failure, pioglitazone, was included in this section (8). On the other hand, other studies have also found no statistically significant differences in the occurrence of MACE, all-cause mortality or heart failure hospitalisation between pioglitazone and the placebo or active control groups (9). This contradictory result indicates that pioglitazone has diverse effects on the heart, and therefore, the heart failure associated with the drug is not a single clinical manifestation but rather the result of several pathophysiological changes induced by PPAR*γ*, some of which may be in opposition to each other (10).

Pioglitazone-related heart failure is not only a result of an increase in fluid volume but also caused by various direct damage to the heart muscle, changes in the whole-body metabolism and blood vessels, and neurohormonal activation. Preclinical and clinical studies show that pioglitazone may directly protect the heart by inhibiting the development of atherosclerosis and in-stent restenosis, slowing down the advance of atrial fibrillation, and consistently improving the electrophysiological properties, energy metabolism, ischemia-reperfusion injury and cardiac remodeling of cardiomyocytes (2). Pioglitazone reduces hypoxia-induced apoptosis of cardiomyocytes in animal models of myocardial ischemia-reperfusion injury by inhibiting the NLRP3/caspase-1 signalling pathway (11). Activation of PPAR*γ* through Pioglitazone in non-human primate models of cardiac sympathetic neurodegeneration helps preserve cardiac sympathetic innervation (12). Recently, *in vitro* toxicity studies using human-derived cardiomyocytes have also identified possible cardiotoxic mechanisms of pioglitazone. Toxicoproteomic analysis showed that pioglitazone exposure leads to severe mitochondrial dysfunction via reduced oxidative phosphorylation and activates a specific cell death pathway for mitochondrial-mediated necroptosis (5). In a related toxicometabolomics study, TZDs acutely inhibited the mitochondrial carnitine shuttle system and altered purine and amino acid metabolism (6).

Renal fluid retention in pioglitazone and the rise in plasma volume due to this have been identified as factors contributing to heart failure. The increased workload of this section can cause or worsen heart failure in people who already have heart problems or a reduced capacity for cardiac output. Drugs are also able to modify blood vessels and regulate neurohormones in the process. The final clinical results will be affected by the patient's initial heart disease and other diseases, the dose of pioglitazone, etc.

At present, given the new treatment system for diabetes and the reduced side effects of some other new drugs, pioglitazone is now being used alongside these other medications. Pioglitazone and Sodium-Glucose Co-transporter 2 Inhibitors (SGLT2i) are now known to be used together. Based on the above research, Ipragliflozin reduces fluid retention and heart disease risk factor changes in non-diabetic adults with Type 2 diabetes and hypertension (13). Clinical real-world data have shown that, compared with monotherapy or non-use of these drugs, the combination of pioglitazone and SGLT2 inhibitors is associated with a reduced risk of heart failure and MACE. A few other studies have also reached the same conclusion (14, 15). The combined effect of the above measures has reduced the damage caused by fluid retention in hyperglycaemic heart disease patients taking pioglitazone. This paper will introduce the main pathogenic mechanisms of pioglitazone-induced heart failure, including direct myocardial effects, metabolic and vascular remodelling, renal fluid retention and neurohormonal activation, etc., to provide a comprehensive theoretical basis for the rational use of pioglitazone in patients with heart failure.

### 1.1 Literature search strategy

This review is a narrative review rather than a systematic review; therefore, the literature search was designed to be comprehensive but not exhaustive, aiming to identify key mechanistic and clinical studies across the field. Following an organised search in the PubMed and Web of Science databases, we reviewed relevant papers published within the past six years leading up to this time. Search terms included combinations of “pioglitazone”, “thiazolidinedione”, “heart failure”, “cardiotoxicity”, “fluid retention”, “PPAR*γ*” and “SGLT2 inhibitor”, and “neurohormonal activation”. Inclusion criteria were: original research articles, clinical trials, meta-analyses and mechanistic studies; English language; and relevance to pioglitazone and cardiovascular outcomes. Exclusion criteria were conference abstracts, non-English papers, and case reports; however, rare adverse events reported in the literature were included. The first search returned more than 800 papers. Screening the titles and abstracts, removing duplicates, and then several hundred articles were read in full. A large number of studies met the inclusion criteria and were included in this review. Evidence strength grading throughout the review: High indicates that multiple randomized controlled trials or meta-analyses have shown consistent results; Moderate means that there is consistent evidence from animal models or prospective cohort studies but limited human RCT data; Limited refers to studies that are mainly *in vitro* experiments, indirect evidence from non-cardiac tissues, or those with conflicting results.

## 2 Mechanism of PPAR*γ* activation and renal fluid retention

### 2.1 How pioglitazone causes fluid retention begins in the renal collecting duct

Pioglitazone is a TZD-class PPAR*γ* agonist that activates PPAR*γ* in the principal cells of the renal collecting duct to promote the expression of the epithelial sodium channel (ENaC). Therefore, more sodium and water are reabsorbed into the blood. The mechanism of fluid retention is not solely reduced sodium reabsorption; other reasons for changes in vascular permeability and neurohormonal activation will also be explained in the following sections.

Preclinical studies provide direct evidence for this pathway. In mouse models, pioglitazone restores the diurnal expression rhythm of core clock genes in the renal medulla, and simultaneously increases the mRNA expression of Scnn1 g encoding the ENaC *γ* subunit, which is associated with enhanced sodium reabsorption and reduced urinary sodium excretion (16). Consistently, studies in type 2 diabetic mice with non alcoholic steatohepatitis NASH found that repeated pioglitazone administration significantly upregulates the mRNA expression of ENaC *α*, *β* and *γ* subtypes in the renal medulla by 1.5 to 2 times, functionally correlated with decreased plasma osmotic pressure and an imbalance between water intake and urine volume (13).

Clinically, TZDs are contraindicated in patients with New York Heart Association (NYHA) class III/IV heart failure precisely due to this sodium-retaining effect (17). Importantly, the sodium-retaining effect induced by PPAR*γ* activation is not unique to synthetic agonists such as pioglitazone. Studies on oxidized phospholipids, such as azPC, which also act as PPAR*γ* agonists, confirmed that they rapidly stimulate sodium transport in the proximal tubule through a non-genomic PPAR*γ*-dependent pathway involving CD36 and ERK signaling (18).

Osmotic diuretics are SGLT2 inhibitors that can address this side effect to some extent. Ipragliflozin is a SGLT2 inhibitor that reduces fluid accumulation and heart enlargement in mouse models caused by pioglitazone without altering ENaC expression. Clinical studies of patients with type 2 diabetes have also shown that, when combined with monotherapy for pioglitazone, dapagliflozin or empagliflozin (SGLT2 inhibitors), the hypervolemia induced by pioglitazone can be reduced significantly (19). Empagliflozin is an SGLT2 inhibitor that can also reduce fluid accumulation caused by TZDs by protecting the vascular integrity of white adipose tissue and inhibiting vascular leakage through the VEGF-A/VEGFR2 signalling pathway (20).

### 2.2 Alterations in vascular permeability and plasma volume expansion

PPAR*γ* activation induced by TZDs is a core mechanism leading to fluid retention and plasma volume expansion. A key initiating event is the direct regulatory effect of PPAR*γ* agonists on vascular endothelial barrier function (21, 22). Mechanistic reviews have pointed out that PPAR*γ* activation increases vascular endothelial permeability and promotes vascular endothelial growth factor (VEGF) secretion, accelerating fluid extravasation from the intravascular space to the interstitial space (7). The resulting reduction in effective circulating volume triggers compensatory neurohormonal activation, and the renin-angiotensin-aldosterone system (RAAS) and antidiuretic hormone (ADH) system are reflexively activated to maintain perfusion pressure by increasing renal sodium and water reabsorption (23).

The two reasons are increased vascular permeability and renal water reabsorption; therefore, there is a relatively fast and large increase in intravascular volume. An increase in preload will increase the work done by the heart. Patients with heart disease or structural problems in the heart are more likely to suffer acute decompensated heart failure or a worsening of chronic stable heart failure due to sudden volume overload.

## 3 Direct negative impacts on myocardial structure and function

Based on the results of the previous studies, we have now observed both a protective and harmful impact on the heart by pioglitazone. Table 1 is as follows.

**Table 1:** Dual evidence on direct myocardial effects of pioglitazone.

Evidence direction	Key findings	Strength
Protective	Inhibits NLRP3/caspase-1 pathway, reduces hypoxia-induced cardiomyocyte apoptosis	M
Protective	Maintains cardiac sympathetic innervation (non-human primate model)	M
Protective	Ameliorates doxorubicin-induced cardiotoxicity	M
Harmful	Induces mitochondrial dysfunction, impairs oxidative phosphorylation (human cardiomyocytes)	L
Harmful	Disrupts cytoskeletal architecture via integrin signaling → contractile failure	L
Harmful	Disturbs purine and amino acid metabolism associated with hypertrophy	L
Harmful	Inhibits CISD1, increases ferroptosis susceptibility	L
Context-dependent	Improves mitochondrial function in diabetes models; induces dysfunction in obesity models	M

M, moderate evidence strength; L, limited evidence strength.

### 3.1 Cardiomyocyte hypertrophy and pathological remodeling

Pioglitazone has various regulatory functions for cardiomyocytes. *In vitro* experiments with neonatal rat cardiomyocytes showed that pharmacological activation of PPAR*γ* by pioglitazone promotes both cardiomyocyte proliferation and hypertrophy (24). Under pre-existing cardiac stress, this will likely lead to maladaptive hypertrophy. Toxicometabolomics shows that when pioglitazone is given acutely, purine metabolism is disrupted, and amino acid levels of L-tyrosine and valine required for cardiac hypertrophy are reduced.

In the heart under stress or in the presence of volume overload, pioglitazone's pro-hypertrophic effect may worsen adverse ventricular remodelling. Based on the clinical data, pioglitazone has been linked to an elevated risk of heart failure hospitalisation in many randomised controlled trials and meta-analyses. This risk is dose-dependent; therefore, a higher HF risk is associated with a dose of 45 mg/day of high-dose pioglitazone compared to a lower dose (25).

Animal studies have shown that pioglitazone is able to induce structural modifications in pathological hypertrophy directly. Mouse models of type 2 diabetes mellitus (T2DM) show that traditional thiazolidinediones (TZDs) such as pioglitazone cause weight gain, fluid retention and cardiac hypertrophy; a new small-molecule inhibitor of PPAR*γ* acetylation was found that increased insulin sensitivity without these side effects, suggesting that the hypertrophic effects are specific to traditional PPAR*γ* agonism (26).

### 3.2 Impaired myocardial contractility

The primary pathological mechanism of myocardial contractile dysfunction in pioglitazone-induced heart failure is a direct negative inotropic effect and disturbances in cardiomyocyte energy metabolism. The source of the strength of evidence for this mechanism is mainly *in vitro* experiments, and clinical data are scarce.

Studies on human-derived cardiomyocytes *in vitro* have revealed the mechanism of this cardiotoxicity. Comprehensive toxicoproteomic analysis toxicoproteomic analysis showed that pioglitazone is reduced by lowering mitochondrial ATP production. Proteomics experiments have identified that a disruption of cytoskeletal organisation due to altered integrin-mediated signals is a primary cause of reduced heart muscle function in the presence of pioglitazone (5). Research in rats has also shown that a lack of glutathione can cause severe problems with heart muscle contraction, such as reduced force-of contraction and changes in how calcium is regulated inside the cell (27).

The degree of negative inotropy in borderline cardiac function is more serious. In these groups of people, the additional damage to the heart's contractility and energy supply caused by pioglitazone may exceed its reserve, resulting in new-onset heart failure (28).

Pioglitazone, a PPAR*γ* agonist, activates three interconnected pathways that converge on heart failure: renal mechanisms (high evidence strength) characterized by ENaC upregulation, increased sodium and water reabsorption, and plasma volume expansion; direct myocardial effects (limited evidence strength) including cardiomyocyte hypertrophy, impaired contractility, and mitochondrial dysfunction; and vascular and neurohormonal mechanisms (moderate evidence strength) involving vasodilation, RAAS activation, and sympathetic nervous system activation. The final common pathway is heart failure predominantly presenting as volume overload. Evidence strength grading: high (multiple RCTs or meta-analyses), moderate (consistent animal or cohort studies), limited (primarily *in vitro* studies) (Figure 1).

**Figure 1:**
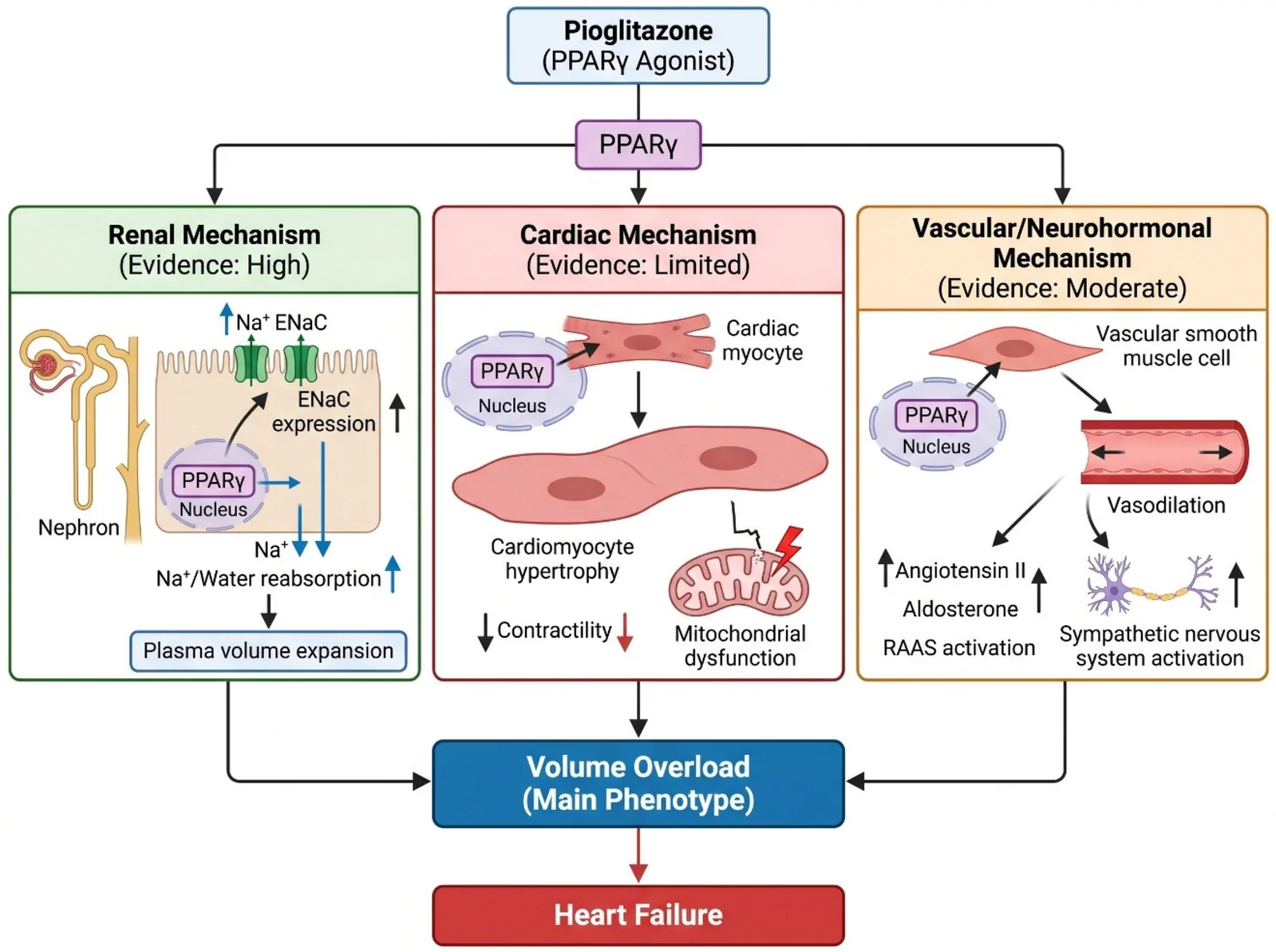
Schematic illustration of the core pathophysiological mechanisms linking pioglitazone to heart failure.

## 4 Myocardial energy metabolic remodeling and dysfunction

### 4.1 Imbalance in myocardial substrate utilization from fatty acids to glucose

As a high-affinity PPAR*γ* agonist, pioglitazone improves whole-body insulin sensitivity and thus reduces glucose levels in hyperglycaemia (29, 30). However, this systemic metabolic reprogramming exerts unique and potentially harmful effects on myocardial metabolism. Pioglitazone-mediated cardiac PPAR*γ* activation may excessively inhibit myocardial fatty acid oxidation. The failing heart is characterized by reduced metabolic flexibility; pioglitazone-induced metabolic remodeling may further aggravate this inflexibility (31). Multi-omics studies on TZD cardiotoxicity support the above view by identifying metabolic disorders in cardiomyocytes, such as reduced oxidative phosphorylation and compensatory up-regulation of anaerobic glycolysis (32). A reduction in efficient fatty acid oxidation has led to a relatively less favourable state of energy supply and demand in the heart muscle. Therefore, an imbalance in myocardial substrate utilisation can lead to serious pathological reasons for a metabolically ill heart to develop overt heart failure.

### 4.2 Mitochondrial dysfunction

Pioglitazone inhibits the mitochondria in some way and thus is associated with heart failure. The research results in the literature are inconsistent and highly context-dependent.

Protective Evidence: Pioglitazone promotes PGC-1*α* expression in diabetic rabbit models, increases mitochondrial biogenesis and dynamics, and suppresses atrial fibrillation (33). Pioglitazone reduces mitochondrial oxidative stress and inflammation in myocardial ischemia-reperfusion injury (34). Pioglitazone is a PGC-1*α* activator that promotes mitochondrial biogenesis in the rat model of prenatal hypoxic-ischemic brain injury (35). Pioglitazone ameliorates the disorder of mitochondrial proteins in skeletal muscle oxidative phosphorylation in insulin-resistant humans ^(^36)

Harmful evidence: Pioglitazone induces whitening in brown adipose tissue of diet-induced obese mice and is associated with impaired thermogenesis and mitochondrial dysfunction **(**37). Pioglitazone in human cardiomyocytes reduces mitochondrial ATP production significantly. Isolated rat hearts have been used to show that both rosiglitazone and pioglitazone cause dose-dependent increases in mitochondrial oxidative stress independent of PPAR*γ* **(**38).

Synthesis: Although pioglitazone can improve mitochondrial function under some conditions of metabolic stress, such as diabetes, off-target or context-specific adverse effects may cause severe damage to cardiomyocyte mitochondrial integrity through direct mitochondrial uncoupling, energy metabolism disorders and oxidative stress in vulnerable areas, leading to energy deficiency, cell death and structural remodeling.

## 5 Effects on the vascular system and afterload

### 5.1 Systemic vasodilation and baroreflex activation

Pioglitazone is a vasodilator that activates multiple pathways to reduce vascular tone through the action of PPAR*γ* (39, 40). Although this reduced afterload theoretically lowers the heart's work, a compensatory neurohormonal response occurs: reduced arterial pressure stimulates arterial baroreceptors and thus increases sympathetic nervous system outflow (41). Gestational RAS modulator exposure also remodels the endotoxic cardiovascular response in adult offspring and thus developmental programming of vascular reactivity (42). Sustained elevation of sympathetic activity can cause damage to the heart over time and be especially harmful to hearts already under stress or damaged. Sustained sympathetic activation leads to damage in the heart muscle through apoptosis and thus causes irregular heart rhythms, thickening and scarring of the heart. Therefore, if the initial beneficial vasodilation induced by pioglitazone is not properly regulated in terms of reflexive neurohormonal activation, myocardial damage and the progression of heart failure may occur.

### 5.2 Pulmonary vascular reactivity and right ventricular afterload

A few case reports and studies have shown that pioglitazone may reduce pulmonary vascular reactivity. However, it is an uneven distribution. Several models of pulmonary hypertension have been used to research pioglitazone's inhibitory effect on the severity of PH and reduction of mean pulmonary arterial pressure; hypoxia-induced PH and monocrotaline-induced PH are representative examples (43, 44). These protective effects are mediated by multiple mechanisms including restoring pulmonary endothelial insulin sensitivity, inhibiting PDGF-induced PASMC proliferation and migration, and modulating the gut microbiome (45, 46), and reversing SERCA2 dysfunction-induced pulmonary vascular remodeling via PPAR*γ* activation (47). High Pulmonary Artery Pressure increases Right Ventricular Afterload directly. However, the application of the results of these experiments in clinical practice has not been realised yet, and fluid retention induced by pioglitazone remains a significant problem in the clinic. The effect of the above drug on the right ventricle of patients with pre-existing pulmonary vascular disease has not been fully studied.

## 6 Activation of the neurohumoral system and the vicious cycle

### 6.1 Compensatory RAAS and sympathetic nervous system activation

As shown above, a relative reduction in the effective arterial blood volume caused by fluid accumulation and vasodilation serves as the initial trigger for neurohormonal compensation, including RAAS and SNS. Pioglitazone-mediated activation of PPAR*γ* enhances the transcription of the renin gene and thus promotes RAAS activation (48). The following elevations of angiotensin II and norepinephrine directly cause cardiovascular damage by intensifying vasoconstriction, increasing afterload, and inducing pathological changes in the heart such as myocardial hypertrophy, fibrosis, and apoptosis; thus, adverse cardiac remodelling occurs (49).

This mechanistic pathway is supported by meta-analyses showing that pioglitazone use is associated with a significantly increased risk of HF and hospitalization for heart failure. A meta-analysis of randomized controlled trials indicated that pioglitazone increased the odds of heart failure by 47% and hospitalization for heart failure by 48% compared to controls (4).

### 6.2 Increased secretion of antidiuretic hormone (arginine vasopressin)

ADH in response to changes in blood volume and osmolality. Given the situation of pioglitazone-induced fluid retention, prolonged action of ADH on renal collecting duct V2 receptors increases water reabsorption by promoting aquaporin-2 membrane translocation and thus exacerbating water retention. In addition to the above renal effects, ADH is also a known vasoconstrictor in the vascular smooth muscle cells of the V1a receptor pathway, which increases afterload. An increase in preload due to fluid accumulation and a decrease in afterload from vasoconstriction together reduce cardiac output significantly more so in people with heart failure.

## 7 Regulation of myocardial inflammation and fibrosis progression

### 7.1 The paradox of PPAR*γ* in heart failure: when systemic anti-inflammation fails the heart

PPAR*γ* has demonstrated powerful anti-inflammatory functions in many parts of the body and has been frequently identified in studies of neuroinflammation, kidney damage and damage to the inner ear (50–55). This effect is generally considered cardioprotective, and indeed pioglitazone has shown benefit in doxorubicin-induced cardiotoxicity and myocardial ischemia-reperfusion injury (56, 57). However, in the special case of heart failure with preserved ejection fraction (HFpEF) or diabetic cardiomyopathy, the same anti-inflammatory characteristics may be counterproductive.

PPAR*γ* activation of cardiac fibroblasts under conditions of metabolic stress or pressure overload may paradoxically promote TGF-*β*/Smad-mediated fibrosis, which is a primary cause of diastolic dysfunction. Therefore, cell-type and context-dependent effects are observed, and systemic anti-inflammatory benefits in macrophages may be outweighed by local pro-fibrotic signals in the myocardial interstitium. Clinically, although pioglitazone has demonstrated systemic anti-inflammatory effects, it has not proven effective in improving outcomes in diastolic heart failure; in fact, some studies suggest that systemic anti-inflammatory approaches may paradoxically increase myocardial fibrosis in patients with HFpEF. For instance, preclinical evidence indicates that PPAR*γ* activation in cardiac fibroblasts promotes myofibroblast differentiation and increases collagen type I and III synthesis, directly contributing to interstitial fibrosis and diastolic dysfunction. Directly promoting the pro-fibrotic effects of key effector cells in the process of extracellular matrix remodelling can be considered a possible mechanism by which pioglitazone promotes interstitial fibrosis. Clinically, there is severe ventricular mechanical dysfunction, and diastolic dysfunction is the main type, which is typical for HFpEF (58)^.^

## 8 Molecular basis of individual susceptibility differences

### 8.1 Influence of genetic polymorphism

Genetic variations in genes for proteins that affect the pharmacokinetics and pharmacodynamics of pioglitazone have led to various degrees of inter-individual differences in drug concentration and response. Among these, polymorphisms in cytochrome P450 2C8 (CYP2C8) are relatively common and significantly affect the metabolic clearance rate of pioglitazone. For example, the CYP2C8*3 allele has increased metabolic clearance of thiazolidinediones. Genetic variations that affect drug transporters, such as those of organic anion-transporting polypeptide 1B1 (OATP1B1), can also alter the plasma and tissue concentrations of PPAR*γ* agonists. Polymorphisms in the drug's direct target (the PPAR*γ* receptor) or in the downstream signaling pathway may increase the susceptibility of some patients to myocardial and renal damage from pioglitazone, as well as other pharmacokinetic reasons. It has many applications in practice. Drug interaction studies have shown that a potent CYP2C8 inhibitor, gemfibrozil, increases the AUC of pioglitazone by as much as three times; therefore, this elevated drug exposure and a higher risk of toxicity, including fluid-related problems, need to be managed (59).

### 8.2 Superimposed effects of underlying disease states

People with other illnesses are more likely to develop heart failure after taking pioglitazone because a weakened reserve of the body may be unable to compensate for the sodium- and water-retaining properties of pioglitazone itself. Patients with subclinical or overt left ventricular dysfunction, chronic kidney disease (CKD), advanced age and/or concurrent use of non-steroidal anti-inflammatory drugs all have reduced physiological adaptive capacity (60).

In this group of vulnerable people, mild-to-moderate expansion of plasma volume induced by pioglitazone can act as a powerful “second blow” and rapidly cause clinically significant heart failure in the presence of pre-existing heart disease. Clinical cases of elderly patients or those with multiple diseases who developed severe oedema, anasarca and even pleural effusion after taking pioglitazone are also provided above.

Therefore, according to the clinical practice guidelines, pioglitazone is generally not recommended for symptomatic heart failure (NYHA Class III or IV), and it should be used with caution by individuals who are high-risk, such as those with a history of left ventricular dysfunction or chronic kidney disease (CKD) (61).

## 9 Insulin status and hypoglycemia as contributing factors (brief overview)

Insulin has a weak antidiuresis and anti-sodium retention effect. Pioglitazone increases insulin sensitivity, and in the presence of insulin secretagogues, a relative or absolute rise in insulin may occur, thus promoting renal sodium reabsorption (62). However, this effect is weak compared with direct PPAR*γ*-mediated ENaC upregulation. Severe hypoglycemia is a primary risk of combination therapy for pioglitazone and sulfonylureas or insulin; it causes catecholamine release and an increased oxygen demand in the heart muscle, potentially leading to ischaemia or arrhythmia in susceptible individuals (63–65). From a clinical perspective, when pioglitazone is used in conjunction with other drugs, especially in older adults or people with a history of coronary heart disease, it is necessary to limit the hypoglycemic effect of co-administered drugs as much as possible to prevent cardiovascular stress caused by catecholamines. The following are additional studies on the identification of high-risk patients.

## 10 Clinical implications and future research directions

### 10.1 Identification and risk stratification of high-risk patients

Based on the mechanistic studies and clinical data, both suggest that high-risk patients should undergo screening before being treated with pioglitazone. At the bottom of risk stratification is the assessment of the patient's original heart function and volume status. Patients with pre-existing heart failure, including those in a subclinical state, are a very high-risk group. A large retrospective cross-sectional study of NT-proBNP to identify subclinical heart failure in ambulatory T2DM patients found that a positive screen was significantly associated with pioglitazone use. CKD patients are also a group at high risk (66). Patients with hypertension or volume overload who have already received the highest doses of RAAS inhibitors or diuretics are at an even higher risk. Severe hypoglycemia when taking pioglitazone and sulfonylureas or insulin alone predicts an increased risk of cardiovascular disease and death in older people and those with many other risks for cardiovascular disease (67). Perform a comprehensive pre-therapy assessment, obtain a complete medical history of heart failure symptoms, conduct a physical examination for oedema, measure renal function, and, in the case of older patients with a history of diabetes or other known cardiovascular diseases, measure natriuretic peptides such as BNP or NT-proBNP and perform echocardiography (68).

This algorithm integrates the mechanistic insights discussed in the text into a practical clinical framework. Patients being considered for pioglitazone therapy undergo initial screening for high-risk features, including symptomatic heart failure (NYHA class III/IV), chronic kidney disease, pre-existing left ventricular dysfunction, or concomitant use of insulin/sulfonylureas. High-risk patients are contraindicated for or should avoid pioglitazone. Low-risk patients may receive pioglitazone with risk mitigation strategies, including low-dose initiation (15–30 mg/day), addition of an SGLT2 inhibitor, or addition of amiloride (ENaC inhibitor), with loop diuretic adjustment as needed. This algorithm is derived from clinical trial evidence, meta-analyses, and mechanistic studies cited in the main text (Figure 2).

**Figure 2:**
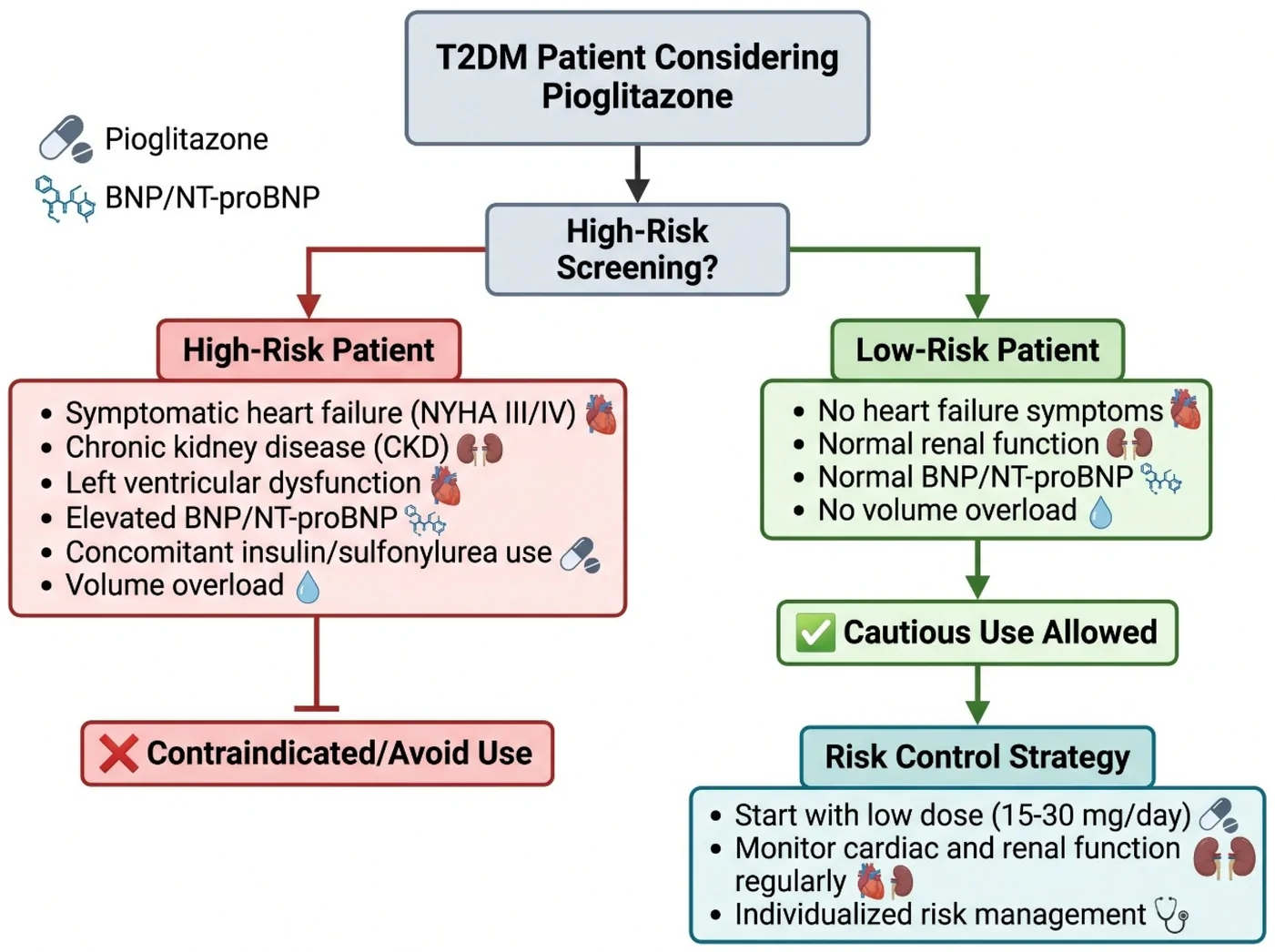
Clinical decision algorithm for risk stratification and management of pioglitazone-associated heart failure.

### 10.2 Adjustment of treatment strategies and drug intervention targets

For patients who require continuous pioglitazone but experience fluid retention, the following clinical management options can be employed: strengthening diuretic therapy and, in some cases, adding an ENaC inhibitor such as amiloride or reducing the dose of pioglitazone. The scientific basis of ENaC inhibition comes from the disease mechanism of fluid retention caused by pioglitazone. The other is a reduced dosage. Based on the data from the IRIS trial, while a daily dose of 45 mg of pioglitazone is effective, other lower doses such as 15 mg or 30 mg per day also reduced the risk of stroke and myocardial infarction significantly, and had a better safety profile regarding edema, weight gain and heart failure. This dose-response benefit-risk profile was further supported by the primary publication of the IRIS trial (69). Given that these patients are at an increased risk of fluid overload, it is recommended to reduce pioglitazone gradually to the minimum effective dose of 15–30 mg/day and add standard or intensified loop diuretic therapy. Based on the mechanism, adding an ENaC inhibitor such as amiloride may serve as an adjunct to the diuretic therapy for these patients, but clinical outcome data on the combined use with pioglitazone are still lacking

Future drug development aims to separate the glycemic benefits of PPAR*γ* activation from its detrimental cardiac and renal side effects by designing selective PPAR*γ* modulators or SPPAR*γ*Ms or PPAR*γ* and *δ* dual agonists (70, 71).

### 10.3 Major knowledge gaps and future research prioritie

The primary sources of the missing knowledge are listed below. First, the tissue-specific molecular basis of the different PPAR*γ* effects in the kidney, heart and adipose tissue has not been fully elucidated. Second, no reliable biomarkers or imaging parameters have been identified to predict the progression of subclinical volume expansion into overt heart failure. Thirdly, the safety and effectiveness of selective PPAR*γ* modulators in humans, especially in terms of fluid retention and cardiac outcomes, need to be confirmed clinically. Fourth, in the future, series of NT-proBNP and cardiac imaging studies will be conducted to monitor subclinical changes under pioglitazone treatment. Fifthly, the function of genetic polymorphisms in CYP2C8, ENaC and PPAR*γ* as predictors for pioglitazone-related heart failure needs to be verified prospectively. Finally, it has not been determined when to start an SGLT2 inhibitor in relation to pioglitazone for maximum cardioprotection.

### 10.4 Clinical translation: from mechanisms to bedsid

Table 2

**Table 2:** Clinical translation of mechanistic insights.

Mechanism	Clinical implication	Actionable recommendation	Strength	Gap
ENaC upregulation	Fluid retention refractory to loop diuretics	Consider amiloride add-on therapy	M (mechanism)/L (clinical)	No RCT for this indication
Volume expansion	Increased preload precipitates HF in vulnerable patients	Screen with BNP or NT-proBNP before initiation	H	Threshold values not established
SGLT2 counter-regulation	Osmotic diuresis opposes TZD retention	Prioritize pioglitazone plus SGLT2i combination	H (real-world)/L (RCT)	No dedicated RCT
Dose-dependent effects	Lower doses retain benefits with less fluid retention	Use 15 to 30 mg per day as starting dose in at-risk patients	H (post-hoc)	Prospective dose-comparison trials needed
Genetic polymorphisms	Variable drug clearance and response	Consider CYP2C8 genotyping in selected patients	M	Cost-effectiveness unknown

H, high evidence strength supported by multiple randomized controlled trials or meta-analyses with consistent findings; M, moderate evidence strength supported by consistent evidence from animal models or prospective cohort studies with limited human RCT data; L, limited evidence strength primarily derived from *in vitro* studies, indirect evidence from non-cardiac tissues, or studies with conflicting results.

### 10.5 Evidence limitations and translational gaps

Some serious defects in the present foundation of evidence need to be rectified. Many cardiotoxic mechanisms proposed thus far have been derived from human cardiomyocyte cell lines (5). The above results need to be validated in animal experiments and then on people. Rodent models do not fully replicate the cardiac physiology of humans, and PPAR*γ* expression patterns and susceptibility to fluid retention are also altered. Some of these mechanisms have been identified in studies of the kidney, fat, and liver, but they may not be directly applicable to the heart (37). Most of the mechanisms mentioned have not been the subject of specific prospective human studies to test their mechanistic hypotheses. The literature may be more likely to publish harmful results that are more likely to be published than null or protective findings. Based on the above deficiencies, readers should be cautious about accepting mechanistic explanations.

## 11 Conclusion

Pioglitazone and heart failure have the opposite effects, and their combination is therefore likely not beneficial. At the same time, the listed medications have certain positive effects; they can help enhance the sensitivity of insulin, decrease inflammation and slow the progression of atherosclerosis to reduce the risk of a heart attack or stroke in high-risk patients. At the same time, there are harmful effects, such as PPAR*γ*-mediated renal sodium and water retention, increased vascular permeability, and in some experimental contexts, direct myocardial damage that reduces contractility or promotes fibrosis.

The clinical results of whether a patient develops or experiences an aggravation of heart failure depend on the balance among these forces and are heavily affected by various factors that make an individual more susceptible to heart failure, such as previous heart problems, chronic kidney disease, age, genetics, other medications taken, etc.

Most patients with type 2 diabetes who are hemodynamically stable have a low absolute risk of heart failure with pioglitazone, and the cardiovascular benefits of reduced MACE and stroke may outweigh the risks. However, for patients with severe heart failure, especially those in NYHA Class III or IV, chronic kidney disease, and other risk factors for volume overload, pioglitazone is contraindicated or should be used very cautiously.

This nuanced view, rejecting both uniform cardiotoxicity and ignorable risk, supports a stratified approach: pioglitazone is safe for most T2DM patients with normal cardiac and renal function, but contraindicated in those with symptomatic HF or advanced CKD. Future research must prioritize tissue-specific PPAR*γ* targeting and prospective validation of risk prediction tools, including genetic and biomarker-based strategies.

## References

[1] Bell DSH Jerkins T. In praise of pioglitazone: a multifaceted insulin sensitiser. Diabetes Obes Metab. (2023) 25:3093–102. 10.1111/dom.15222 37534526

[2] Nesti L Tricò D Mengozzi A Natali A. Rethinking pioglitazone as a cardioprotective agent: a new perspective on an overlooked drug. Cardiovasc Diabetol. (2021) 20:109. 10.1186/s12933-021-01294-7 34006325 PMC8130304

[3] Sheikh IM Hassan OA Adam SM Ali AI Ogedegbe OJ Tabowei G. Association of pioglitazone with major adverse cardiovascular events and heart failure: a systematic review and meta-analysis. Cureus. (2023) 15:e46911. 10.7759/cureus.46911 37954768 PMC10639032

[4] Sinha B Ghosal S. Assessing the need for pioglitazone in the treatment of type 2 diabetes in the context of cardiovascular outcome trials: a meta-analysis. Sci Rep. (2020) 10:15781. 10.1038/s41598-020-72967-8 32978507 PMC7519682

[5] Sultan AA Rattray Z Rattray NJW. Cytotoxicity and toxicoproteomics analysis of thiazolidinedione exposure in human-derived cardiomyocytes. J Appl Toxicol. (2024) 44:1214–35. 10.1002/jat.4613 38654465

[6] Sultan AA Rattray Z Rattray NJW. Toxicometabolomics-based cardiotoxicity evaluation of thiazolidinediones in human-derived cardiomyocytes. Metabolomics. (2024) 20:24. 10.1007/s11306-024-02097-z 38393619 PMC10891199

[7] Sinnathamby ES Urban BT Clark RA Roberts LT De Witt AJ Wenger DM. Etiology of drug-induced edema: a comprehensive review. Cureus. (2024) 16:e53400. 10.7759/cureus.53400 38435190 PMC10908346

[8] Zhu J Yu X Zheng Y Li J Wang Y Lin Y. Association of glucose-lowering medications with cardiovascular outcomes: an umbrella review and evidence map. Lancet Diabetes Endocrinol. (2020) 8:192–205. 10.1016/S2213-8587(19)30422-X 32006518

[9] Mannucci E Giaccari A Gallo M Targher G Pintaudi B Candido R. Effects of pioglitazone on cardiovascular events and all-cause mortality in patients with type 2 diabetes: a meta-analysis of randomized controlled trials. Nutr Metab Cardiovasc Dis. (2022) 32:529–36. 10.1016/j.numecd.2021.12.006 35144855

[10] Wijnen M Duschek EJJ Boom H Van Vliet M. The effects of antidiabetic agents on heart failure: a narrative review. Neth Heart J. (2022) 30:65–75. 10.1007/s12471-021-01579-2 34097240 PMC8799792

[11] Zhang H Huang C Zhang D Zhu Y. Pioglitazone protects against hypoxia-induced cardiomyocyte apoptosis by blocking the NLRP3/caspase-1 signaling pathway. Int Heart J. (2022) 63:893–903. 10.1536/ihj.21-404 36104240

[12] Metzger JM Matsoff HN Zinnen AD Fleddermann RA Bondarenko V Simmons HA. Post mortem evaluation of inflammation, oxidative stress, and PPAR*γ* activation in a nonhuman primate model of cardiac sympathetic neurodegeneration. PLoS One. (2020) 15:e0226999. 10.1371/journal.pone.0226999 31910209 PMC6946159

[13] Tahara A. Effects of SGLT2 inhibitor ipragliflozin alone and combined with pioglitazone on fluid retention and cardiac hypertrophy in type 2 diabetic mice with NASH. Eur J Pharmacol. (2021) 901:174076. 10.1016/j.ejphar.2021.174076 33798599

[14] Lo S-C Kornelius E Liao P-L Huang J-Y Yang Y-S Huang C-N. Pioglitazone, SGLT2 inhibitors and their combination for primary prevention of cardiovascular disease and heart failure in type 2 diabetes: a real-world cohort study. Diabetes Res Clin Pract. (2023) 200:110685. 10.1016/j.diabres.2023.110685 37100230

[15] Anson M Henney AE Zhao SS Ibarburu GH Lip GYH Cuthbertson DJ. Effect of combination pioglitazone with SGLT2 inhibitors or GLP-1 receptor agonists on cardiovascular outcomes in type 2 diabetes: a systematic review and meta-analysis. Diabetes Obes Metab. (2024) 26:2606–23. 10.1111/dom.15576 38558280

[16] Izmailova O Kabaliei A Shynkevych V Shlykova O Kaidashev I. PPARG agonist pioglitazone influences diurnal kidney medulla mRNA expression of core clock genes and ENaC subunits in mice. Physiol Rep. (2022) 10:e15535. 10.14814/phy2.15535 36511486 PMC9746034

[17] Vaughan EM Santiago-Delgado ZM. Management of type 2 diabetes mellitus with noninsulin pharmacotherapy. Am Fam Physician. (2024) 109:333–42. PMID: 38648832. 38648832

[18] Mizuno T Satoh N Horita S Tsukada H Takagi M Sato Y. Oxidized alkyl phospholipids stimulate sodium transport in proximal tubules through a non-genomic PPAR*γ*-dependent pathway. J Biol Chem. (2022) 298:101681. 10.1016/j.jbc.2022.101681 35124009 PMC8892145

[19] Unlusoy YB Dizdar OS Gunal Aİ. Effect of SGLT2 inhibitors on thiazolidinedione-induced changes in volume status in patients with type 2 diabetes. Arch Endocrinol Metab. (2025) 69:e240485–8. 10.20945/2359-4292-2024-0485 40704755 PMC12403730

[20] Kim JY Jang H-M Lee H-J Lee AH Kim D-H Kim SG. Sodium-glucose cotransporter 2 inhibitor ameliorates thiazolidinedione-induced fluid retention by protecting white adipose tissue vascular integrity. Diabetes Obes Metab. (2026) 28:1764–77. 10.1111/dom.70356 41366590

[21] Low YL Jin L Morris ER Pan Y Nicolazzo JA. Pioglitazone increases blood-brain barrier expression of fatty acid-binding protein 5 and plasma volume expansion markers. Mol Pharm. (2020) 17:873–84. 10.1021/acs.molpharmaceut.9b01131 31944767

[22] Wei X Zhou Y Song J Zhao J Huang T Zhang M. Hyperglycemia aggravates blood-brain barrier disruption following diffuse axonal injury and increases vascular permeability. Inflammation. (2023) 46:129–45. 10.1007/s10753-022-01716-y 35857154

[23] Susilawati E Levita J Susilawati Y Sumiwi SA. Review of the case reports on metformin, sulfonylurea, and thiazolidinedione therapies: focus on adverse events. Med Sci (Basel). (2023) 11:50. 10.3390/medsci11030050 37606429 PMC10443323

[24] Xuan Q Li J Feng Z Zhu L Jiang T Li H. Modulation of PPAR-α and PPAR-*γ* influences cardiomyocyte growth and cardiac remodeling. IUBMB Life. (2025) 77:e70035. 10.1002/iub.70035 40536211

[25] Spence JD Viscoli C Kernan WN Young LH Furie K DeFronzo R. Efficacy of lower doses of pioglitazone after stroke: a *post-hoc* analysis of the IRIS trial. Diabetes Obes Metab. (2022) 24:1150–8. 10.1111/dom.14687 35253334

[26] Wu D Gong Z He Y Kumar S Eeda V Wan Q. Small molecule inhibitor of PPARγ acetylation promotes insulin sensitization and browning of white adipose tissue with improved safety. bioRxiv : The Preprint Server for Biology. (2025). 10.64898/2025.12.15.694265

[27] Li F-J Fu S Ye H Hu Y-H Chen J Privratsky JR. Metallothionein alleviates glutathione depletion-induced oxidative cardiomyopathy and contractile dysfunction in mice. Am J Pathol. (2024) 194:912–26. 10.1016/j.ajpath.2024.02.009 38417695

[28] Sohn M Frias JP Lim S. Cardiovascular efficacy and safety of antidiabetic agents: a focus on heart failure. Diabetes Obes Metab. (2023) 25:3560–77. 10.1111/dom.15251 37649320

[29] Lee K-C Wu P-S Lin H-C. Pathogenesis and treatment of non-alcoholic steatohepatitis and its fibrosis: role of PPAR*γ* agonists. Clin Mol Hepatol. (2023) 29:77–98. 10.3350/cmh.2022.0237 36226471 PMC9845678

[30] Goto H Takamura T. Metabolic dysfunction-associated steatotic liver disease complicated by diabetes: pPAR*γ* as a therapeutic target. J Obes Metab Syndr. (2025) 34:224–38. 10.7570/jomes25017 40537153 PMC12318698

[31] Xi L Sun H Yang N Wang Q Zhang L Song J. Pioglitazone alleviates *β*1-adrenergic receptor antibody-induced atrial fibrillation susceptibility by improving energy metabolism. Curr Med Chem. (2025) 32:5861–78. 10.2174/0109298673308673240527092317 38818915

[32] Sultan AA Rattray Z Rattray NJW. Integrative analysis of toxicometabolomics and toxicoproteomics data reveals multi-level cardiotoxicity mechanisms of thiazolidinediones. Metabolomics. (2024) 21:1. 10.1007/s11306-024-02201-3 39636558 PMC11621136

[33] Zhang Z Zhang X Meng L Gong M Li J Shi W. Pioglitazone inhibits diabetes-induced atrial mitochondrial oxidative stress and improves mitochondrial dynamics. Front Pharmacol. (2021) 12:658362. 10.3389/fphar.2021.658362 34194324 PMC8237088

[34] Luo J Chen L Zhang X Su Q Zhou X Lian Q. Pioglitazone ameliorates mitochondrial oxidative stress and inflammation in myocardial ischemia-reperfusion injury. Cell Prolif. (2026) 59:e70109. 10.1111/cpr.70109 40767000 PMC12961535

[35] Jia L Wang J Cao H Zhang X Rong W Xu Z. Activation of PGC-1*α* and mitochondrial biogenesis by pioglitazone protects against prenatal hypoxic-ischemic brain injury. Neuroscience. (2020) 432:63–72. 10.1016/j.neuroscience.2020.02.035 32114097

[36] Fiorentino TV Monroy A Kamath S Sotero R Cas MD Daniele G. Pioglitazone corrects dysregulation of skeletal muscle mitochondrial proteins involved in oxidative phosphorylation in insulin-resistant humans. Metab Clin Exp. (2021) 114:154416. 10.1016/j.metabol.2020.154416 33137378

[37] Yu P Wang W Guo W Cheng L Wan Z Cheng Y. Pioglitazone-enhanced brown fat whitening contributes to weight gain and is associated with mitochondrial dysfunction. Exp Clin Endocrinol Diabetes. (2023) 131:595–604. 10.1055/a-2178-9113 37729949

[38] Riess ML Elorbany R Weihrauch D Stowe DF Camara AKS. PPAR*γ*-independent side effects of thiazolidinediones on mitochondrial redox state in isolated rat hearts. Cells. (2020) 9:252. 10.3390/cells9010252 31968546 PMC7017211

[39] Morgaan HA Sallam MY El-Deeb NM El-Gowelli HM El-Gowilly SM El-Mas MM. Pharmacologic and endotoxic reprogramming of renal vasodilatory signaling: role of PPAR*γ*. Sci Rep. (2025) 15:8137. 10.1038/s41598-025-87586-4 40057533 PMC11890745

[40] Abuiessa SA Helmy MM El-Gowelli HM El-Gowilly SM El-Mas MM. Dysregulated ACE/ang II/Ang1–7 signaling provokes cardiovascular and inflammatory sequelae in septic offspring. Eur J Pharmacol. (2022) 936:175344. 10.1016/j.ejphar.2022.175344 36270538

[41] Bakkar N-MZ Mougharbil N Mroueh A Kaplan A Eid AH Fares S. Worsening baroreflex sensitivity on progression to type 2 diabetes and its modulation by pioglitazone. Am J Physiol Endocrinol Metab. (2020) 319:E835–51. 10.1152/ajpendo.00145.2020 32865011

[42] Abuiessa SA Helmy MM El-Gowelli HM El-Gowilly SM El-Mas MM. Gestationally administered RAS modulators reprogram endotoxic cardiovascular responses in adult rat offspring. Naunyn Schmiedebergs Arch Pharmacol. (2025) 398:699–713. 10.1007/s00210-024-03305-2 39046530

[43] Zhang Z Liang Y Mo S Zhao M Li Y Zhang C. Oral administration of pioglitazone inhibits pulmonary hypertension progression by modulating gut microbiome. Physiol Rep. (2025) 13:e70174. 10.14814/phy2.70174 39739369 PMC11685845

[44] Fan F He J Su H Zhang H Wang H Dong Q. Tribbles homolog 3-mediated vascular insulin resistance contributes to hypoxic pulmonary hypertension and is reversed by pioglitazone. Front Physiol. (2020) 11:542146. 10.3389/fphys.2020.542146 33192545 PMC7662151

[45] Hussain T Chai L Wang Y Zhang Q Wang J Shi W. Activation of PPAR-*γ* prevents TERT-mediated pulmonary vascular remodeling in monocrotaline-induced pulmonary hypertension. Heliyon. (2023) 9:e14173. 10.1016/j.heliyon.2023.e14173 36938425 PMC10015197

[46] Zhang Q Feng W Wang Q Wang J Chai L Chen Y. PPAR*γ* activation inhibits PDGF-induced pulmonary artery smooth muscle cell proliferation and migration through PTEN upregulation. Biomed Pharmacother. (2022) 152:113233. 10.1016/j.biopha.2022.113233 35689861

[47] Qiu Y Chen H Xie Y Wang Y Huang X Li L. SERCA2 dysfunction stimulates inflammation and causes pulmonary vascular remodeling: reversal by PPAR*γ* activation. Eur J Pharmacol. (2026) 1012:178502. 10.1016/j.ejphar.2025.178502 41420972

[48] Yang X Qi Y Hao J Wei H Li Z Xu M. Effects of oral antidiabetic agents on the renin-angiotensin-aldosterone system: a systematic review. Eur J Clin Pharmacol. (2025) 81:801–13. 10.1007/s00228-025-03830-w 40167623

[49] Hsu C-N Hsuan C-F Liao D Chang JK-J Chang AJ-W Hee S-W. Anti-diabetic therapy and heart failure: recent advances and mechanistic insights. Life (Basel). (2023) 13:1024. 10.3390/life13041024 37109553 PMC10144651

[50] Haddadi R Cheraghi-Poor M. Peroxisome proliferator activated receptor-gamma (PPAR-*γ*) ligand, pioglitazone, increases analgesic and anti-inflammatory effects in formalin-induced nociception. Naunyn Schmiedebergs Arch Pharmacol. (2024) 397:1633–46. 10.1007/s00210-023-02715-y 37698622

[51] Alhowail A Alsikhan R Alsaud M Aldubayan M Rabbani SI. Protective effects of pioglitazone on cognitive impairment and neuroinflammation in a rat model of Alzheimer’s disease. Drug Des Devel Ther. (2022) 16:2919–31. 10.2147/DDDT.S367229 36068789 PMC9441149

[52] Sekulic-Jablanovic M Wright MB Petkovic V Bodmer D. Pioglitazone ameliorates gentamicin ototoxicity by preserving mitochondrial function. Front Cell Neurosci. (2020) 14:566148. 10.3389/fncel.2020.566148 33192313 PMC7658481

[53] Zhang Y Ruan S Xie Y Rui X Zhou J Wang W. Peroxisome proliferator-activated receptor gamma prevents activation of RBL-2H3 cells via suppressing NF-*κ*B signaling. Inflamm Res. (2025) 74:49. 10.1007/s00011-025-02022-7 40057631

[54] El-Din SS Abd Elwahab S Rashed L Fayez S Aboulhoda BE Heikal OA. Possible role of rice bran extract in microglial modulation through PPAR-gamma receptors in a rat model of neuroinflammation. Metab Brain Dis. (2021) 36:1903–15. 10.1007/s11011-021-00741-4 34043126

[55] Zamanian MY Taheri N Opulencia MJC Bokov DO Abdullaev SY Gholamrezapour M. Neuroprotective and anti-inflammatory effects of pioglitazone on traumatic brain injury: a review. Mediators Inflamm. (2022) 2022:9860855–10. 10.1155/2022/9860855 35757108 PMC9232315

[56] Pakravan G Peymani M Abedpoor N Safaeinejad Z Yadegari M Derakhshan M. Antiapoptotic and anti-inflammatory effects of PPAR*γ* agonist, pioglitazone, reversed doxorubicin-induced cardiotoxicity in rats. J Biochem Mol Toxicol. (2022) 36:e23041. 10.1002/jbt.23041 35343021

[57] Zhang H Lin A Zhai C Lai T Gong X Wang M. Ginsenoside rd promotes cardiac regeneration through PPARG/HMGCS2-driven ketone body metabolic reprogramming. Phytomedicine. (2026) 150:157694. 10.1016/j.phymed.2025.157694 41442992

[58] Li CX Wu RM Xie QL Wang F Xu XL. Procyanidin B2 attenuates pathologic cardiac fibrosis and inflammation: role of PPAR*γ*. J Cardiovasc Pharmacol. (2025) 85:338–49. 10.1097/FJC.0000000000001684 39970127

[59] Jangra A Babu B Divakar S Gowramma B Rajan S Jangra S. An in-depth review of PPAR*γ* modulators as anti-diabetes therapeutics: from full agonists to selective modulators. Drug Metab Rev. (2025) 57:311–37. 10.1080/03602532.2025.2508152 40406962

[60] Gonzalez-Manzanares R Anguita-Gámez M Muñiz J Barrios V Gimeno-Orna JA Pérez A. Prevalence and incidence of heart failure in type 2 diabetes patients: sex-specific differences. Cardiovasc Diabetol. (2024) 23:253. 10.1186/s12933-024-02358-0 39014420 PMC11253346

[61] Katsiki N Kazakos K Triposkiadis F. Contemporary choice of glucose lowering agents in heart failure patients with type 2 diabetes: a focus on pioglitazone. Expert Opin Pharmacother. (2022) 23:1957–74. 10.1080/14656566.2022.2143263 36322877

[62] Mastrototaro L Roden M. Insulin resistance and insulin sensitizing agents: from pathophysiology to clinical practice. Metab Clin Exp. (2021) 125:154892. 10.1016/j.metabol.2021.154892 34563556

[63] Ferhatbegović L Bećirović M Bećirović E Sarajlić S Ribić A Šarić A. Clinical and pathophysiological links between hypoglycemia and cardiovascular risk in patients with diabetes. Front Clin Diabetes Healthc. (2026) 7:1765597. 10.3389/fcdhc.2026.1765597 PMC12916416 41725629

[64] Christou MA Christou PA Kyriakopoulos C Christou GA Tigas S. Effects of hypoglycemia on cardiovascular function in patients with diabetes. Int J Mol Sci. (2023) 24:9357. 10.3390/ijms24119357 37298308 PMC10253702

[65] Huang C Huang L Huang Q Lin L Wang L Wu Y. Mitophagy disorder mediates cardiac deterioration induced by severe hypoglycemia in diabetic mice. Mol Cell Endocrinol. (2023) 575:111994. 10.1016/j.mce.2023.111994 37330037

[66] Joshi A Dalal D Patil S Singh H Hajirnis A Seth C. Screening for occult heart failure in type 2 diabetes mellitus using NT-proBNP: a retrospective cross-sectional study. Cureus. (2024) 16:e72576. 10.7759/cureus.72576 39606498 PMC11602244

[67] Aktas G Tel BMA Tel R Balci B. Treatment of type 2 diabetes patients with heart conditions: a practical approach. Expert Rev Endocrinol Metab. (2023) 18:255–65. 10.1080/17446651.2023.2204941 37078758

[68] Hoca E Kalaycı N Ahbab S Engin İ Ataoğlu HE. The impact of metformin on BNP levels in patients with type 2 diabetes. J Clin Med. (2025) 14:2733. 10.3390/jcm14082733 40283562 PMC12028114

[69] Kernan WN Viscoli CM Furie KL Young LH Inzucchi SE Gorman M. Pioglitazone after ischemic stroke or transient ischemic attack. N Engl J Med. (2016) 374(14):1321–31. 10.1056/NEJMoa1506930 26886418 PMC4887756

[70] Banik A Datta Chaudhuri R Vashishtha S Gupta S Kar A Bandyopadhyay A. Deoxyelephantopin—a novel PPAR*γ* agonist regresses pressure overload-induced cardiac fibrosis and improves cardiac function. Eur J Pharmacol. (2023) 953:175841. 10.1016/j.ejphar.2023.175841 37329972

[71] Fei L Zhao Y. Reassessing the risk-benefit profile of thiazolidinediones in the era of SGLT2 inhibitors and GLP-1 receptor agonists. Endocr Pract. (2025) 31:875–83. 10.1016/j.eprac.2025.04.004 40246234

[72] Jagadish A Notta S Bakeler S Joseph D. Pleural effusion and anasarca associated with pioglitazone: a case report. J Pharm Technol. (2025)41(5):249–52. 10.1177/87551225251348838 40535535 PMC12170548

[73] Chimauriya R Chittal A Malik K DuMont T Haas CJ. Unveiling an uncommon complication: pleural effusion secondary to pioglitazone use. J Community Hosp Intern Med Perspect. (2025) 15:94–8. 10.55729/2000-9666.1541 41789211 PMC12959103

[74] Gan T Wang Q Song Y Shao M Zhao Y Guo F. Canagliflozin improves fatty acid oxidation and ferroptosis in diabetic cardiomyopathy: implications for PPAR*γ* cross-talk. Mol Cell Endocrinol. (2024) 582:112139. 10.1016/j.mce.2023.112139 38128823

[75] Admani B Raza FZ Siddiqui F Ashraf MT Khan MK Habib I. The impact of different antidiabetic drugs on fracture risk and cardiovascular outcomes: a network meta-analysis. Am J Med Open. (2025) 14:100115. 10.1016/j.ajmo.2025.100115 41473904 PMC12745141

